# Examination of Energy Needs and Low Energy Availability Among Historically Black College and University Female Student-Athletes

**DOI:** 10.3390/nu16234160

**Published:** 2024-11-30

**Authors:** Nancy A. Uriegas, Dawn M. Emerson, Zachary K. Winkelmann, Andrew Ortaglia, Toni M. Torres-McGehee

**Affiliations:** 1 Department of Health and Human Performance, College of Education, Texas State University, San Marcos, TX 78666, USA; 2 Department of Exercise Science, Arnold School of Public Health, University of South Carolina, Columbia, SC 29208, USA; 3 Department of Epidemiology and Biostatistics, Arnold School of Public Health, University of South Carolina, Columbia, SC 29208, USA

**Keywords:** female athlete triad, relative energy deficiency in sport, disordered eating

## Abstract

Low energy availability (LEA) is common among female student-athletes and contributes to reduced bone mineral density and hormonal dysfunction. However, limited research exists on student-athletes of color, making it difficult to generalize results across populations, particularly Historically Black Colleges and Universities (HBCUs) student-athletes. Objectives: This study examined the energy needs and LEA prevalence, with or without eating disorder (ED) risk, among HBCU female student-athletes. Methods: Twenty-seven female student-athletes (age = 19 ± 1 year; height = 166.9 ± 7.6 cm; weight = 68.8 ± 11.3 kg) completed baseline measures, including the Eating Disorder Inventory-3 (EDI-3), EDI-3 symptom checklist (EDI-SC), anthropometric measures, and resting metabolic rate (RMR). Participants maintained a dietary log to measure energy intake and wore a heart rate monitor to estimate exercise energy expenditures for 7 days. LEA was defined as <30 kcal/kg of fat-free mass (FFM)/day. Results: The mean energy availability (15.9 ± 10.1 kcal/kg FFM/day) indicated 92.6% of participants (*n* = 25) displayed LEA; of those, 60% (*n* = 15) had LEA with ED risk. ED risk was identified in 59.3% of student-athletes (*n* = 13; EDI-3: *n* = 2; SC: *n* = 3; Both: *n* = 11). Interestingly, of the two student-athletes without LEA, one was identified at risk for EDs using both the EDI-3 and SC. Conclusions: HBCU female student-athletes face high risks of LEA and EDs, with most showing signs of both. This underscores the need for culturally sensitive interventions to improve EA and support mental health in this underserved group. Clinicians should focus on nutrition education and early ED identification to enhance long-term health and athletic performance.

## 1. Introduction

Student-athletes of diverse racial and ethnic backgrounds make up 38% (*n* = 199,166) of the National Collegiate Athletic Association (NCAA) population, with 75,730 being females [1]. While not the majority across NCAA institutions, at Historically Black Colleges and Universities (HBCUs) student-athletes of color make up 92.7% of the population. In addition to NCAA institutions, 30 additional HBCUs are competing through other athletic affiliations (National Association of Intercollegiate Athletics, National Christian College Athletic Association, National Junior College Athletic Association, and United States Collegiate Athletic Association). Through the years, HBCUs have faced financial strain and disadvantages compared to peer institutions. When comparing revenue across NCAA Division I and II regions, predominately White institutions earn more than double the money as HBCUs [2]. Thus, health and performance resources may look differently at HBCUs.

On-campus access to student health services has correlated with better health outcomes and can help students overcome financial obstacles to healthcare access, especially for disadvantaged groups, such as racial and ethnic minorities [3,4,5]. Health centers on campus may play a crucial role in fostering success for HBCU students and student-athletes. However, not all HBCUs offer comprehensive health services, including access to prescribing providers at institution-run clinics. Mueller et al. [6] reported 82.2% of HBCUs had a form of campus health services (i.e., licensed practical nurse, registered nurse or emergency medical technicians, telehealth or contract, or comprehensive health services). However, of these, only 70% offered comprehensive health services with prescribing providers. Specifically for student-athletes, athletic trainers may be the only healthcare provider an athlete sees, and oftentimes, there is a lack of trust in the medical system based on family or personal background and experiences [7]. The NCAA demographic database identified a total of 162 athletic trainers working at HBCU institutions for over 17,000 student-athletes [1]. In comparison, 6007 athletic trainers work for non-HBCUs, which are the majority of NCAA institutions. The ratio of athletic trainers to student-athletes at HBCUs yields 1 to 105 compared to 1 to 84 at non-HBCUs [1]. For student-athletes, athletic trainers may be the first to identify, refer, and treat physical and mental health concerns. Among the many medical concerns student-athletes face, some require immediate identification and collaboration with other medical personnel to diagnose and treat. One example is inadequate nutrition and the associated consequences like the female or male athlete triad (Triad) and Relative Energy Deficiency in Sports (REDs).

In the 1990s, the association between disordered eating (DE), amenorrhea, and osteoporosis was recognized as the female Triad [8,9]. Since then, research has determined there is more than an association between the three named components, and the female Triad was updated to be a spectrum of three interrelated components: energy availability (EA), menstrual status, and bone health [10,11]. Additionally, in 2014, the International Olympic Committee (IOC) introduced a broader term, REDs, which continues to evolve, with updates in 2018 and 2023 [12,13,14]. In the most recent update, the IOC defines REDs as a syndrome of impaired physiological and/or psychological functioning caused by exposure to problematic (prolonged and/or severe) low EA (LEA), consequently resulting in impaired health [14]. Ultimately, the latest models observed in the female Triad [11] and REDs [14] have negative health and performance outcomes centered around LEA and DE patterns. 

In many cases, student-athletes are unaware of the dietary intake required to meet the physiological demands of their physical activity, while others intentionally engage in DE behaviors (restricting/dieting, excessive exercise, purging, etc.) to modify their weight. Low-energy diets achieved intentionally or unintentionally and/or increased exercise expenditure are the most common mechanisms underpinning LEA. Energy availability has an algebraic definition of the difference between energy intake (EI) and exercise energy expenditure (EEE), corrected for fat-free mass (FFM), which reflects the body’s most metabolic tissues [10,15]. For active females, thresholds ≥45 kcal/kg of FFM/day are considered optimal, the range of 30–45 kcal/kg of FFM/day is suboptimal, and those < 30 kcal/kg of FFM/day are deemed low, which can be unsafe and potentially a precursor for detrimental health consequences, including the female Triad and/or REDs [10,15]. 

While the prevalence of LEA among females is highly documented, variations exist across methodologies, as multiple strategies are used to objectively quantify LEA [16]. Participation in sports that are weight-sensitive, esthetic, emphasize leanness, or require heavy amounts of endurance training appears to increase LEA risk. Athletes who engage in endurance activities with high volumes of training oftentimes have high EEE and do not meet the EI to maintain optimal EA [17,18,19]. Females in collegiate sports with emphasis on leanness or esthetics present with prevalence rates ranging from 41 to 96% (cross-country = 41%; track and field = 52% synchronized swimming = 52%; equestrian = 82%; ballet = 96%) [20,21,22,23]. However, females participating in ball or team sports also have a high prevalence of LEA. Among a large sample of female athletes, Torres-McGehee et al. [23] documented that 100% of softball student-athletes had LEA and 82.4% were also at risk for EDs. Other ball or team student-athletes with elevated prevalence of LEA with ED risk were volleyball (70%), beach volleyball (70.6%), and soccer (66.7%) [23]. 

Clinical EDs are characterized by the persistent disturbance of eating or eating-related behaviors, resulting in altered consumption or absorption of food and psychosocial and physical health impairments [24]. Eating disorders are complex and may be rooted in biological, psychological, and social concerns; therefore, considerations of predisposing, precipitating, and perpetuating factors are important [25]. Historically, EDs have been thought of as conditions primarily affecting skinny, white, affluent girls, an acronym known as “SWAG” [26]. In fact, among college students, being female, White, and from an affluent socioeconomic background is highly associated with a perceived need for treatment, diagnosis, and receiving treatment in the past year for an ED [27]. However, the authors state it could also be thought of as students of color being less likely to receive any diagnosis of EDs. Earlier studies report there is a smaller likelihood to seek treatment for EDs among ethnically diverse women; and when they do seek treatment, it is also less likely to be diagnosed or referred for further evaluation [28]. Not receiving a diagnosis or referral could then delay treatment and/or increase the ED severity.

Across sports, DE behaviors and clinical EDs are highly prevalent among female athletes, who often feel pressured to maintain a lean and ideal body [29]. Of concern, athletes engage in DE behaviors at young ages, with a mean dieting age of 13.2 years in females, and an ED risk of 14% [30]. Similarly, high school female athletes of ethnic backgrounds have been classified with DE (African American = 19.2%, Caucasian = 18.4%, Latina = 23.3%) and primarily engage in binge eating and vomiting [31]. In the early 2000s, Bratland-Sanda and Sundgot-Borgen [32] reported the prevalence of DE and EDs across female athletes from adolescence to adulthood ranging from 6 to 45%. To date, findings appear to remain consistent, as nearly 30% of U.S. collegiate student-athletes were at risk for EDs [33]. Notably, female athletes participating in endurance (i.e., cross-country, swimming) accounted for the majority of those at risk for EDs; however, female athletes across all sports categories are at risk [33]. At times, athletes believe engaging in DE behaviors will help them maintain a low body weight and; therefore, improve their performance (i.e., jump higher, run faster). However, these behaviors have significant implications that can ultimately result in negative health consequences and decreased performance, which is the opposite effect they seek. 

Diversity exists across race/ethnicity, socioeconomic status, weight, sex, and gender among individuals presenting with EDs symptoms and/or engaging in pathogenic behaviors [34,35]. Previously, women of color have reported elevated levels of binge eating, dieting, and unhealthy weight control behaviors [36]. In turn, these DE behaviors can play a significant role in LEA across this population. Existing research on the prevalence of LEA, the female Triad, and REDs have primarily been conducted on White–Caucasian females [18,20,22,23,37,38,39,40,41,42]. Limited research exists among female athletes of color, which can pose challenges to the transferability of results. Therefore, the objective of this study was two-fold: (1) to examine the energy needs and nutritional profiles of female student-athletes attending HBCUs and (2) to examine the prevalence of LEA with or without ED risk. We hypothesize that female student-athletes attending HBCUs will not meet the recommended nutritional values for carbohydrates and protein and that a large majority of the female student-athletes will have LEA.

## 2. Materials and Methods

### 2.1. Study Design and Participants

This was a descriptive, cross-sectional study in a free-living environment. G*Power software (version 3.1.94, Heinrich Heine University, Dusseldorf, Germany) was used to calculate the power using chi-square analysis for LEA risk or ED risk, an alpha of 0.05, and a large effect size of 0.60 [43]. A sample size of 22 female student-athletes was needed for a power of 0.80. To be included, participants had to be at least 18 years old and actively participate in an in-season sport (i.e., volleyball, soccer, basketball) at an HBCU. Exclusion criteria included those not actively participating in sports due to medical illness, orthopedic injury, or a previous ED diagnosis. The study was approved by the University of South Carolina Institutional Review Board (PRO00092173) and all participants provided consent.

A total of 30 female student-athletes from 3 NCAA Division I (i.e., highest level of collegiate competition and athletic performance with larger number of athletic scholarships; median undergraduate enrollment = 9000 students) and II (i.e., second level of competition and athletic performance with less or partial athletic scholarships; median undergraduate enrollment = 2500 students) HBCU institutions volunteered and consented to participate in the study. Of these, 3 participants did not complete their dietary logs accurately. Therefore, their data were excluded, yielding a final sample of 27 participants (age = 19 ± 1 year; height = 166.9 ± 7.6 cm; weight = 68.8 ± 11.3 kg). Overall, participants were primarily Black (85.2%, *n* = 23; Hispanic = 7.4%, *n* = 2; Multi-Ethnic = 7.4%, *n* = 2), lowerclassmen (freshman/1st year 33.3%, *n* = 9; sophomore/2nd year = 25.9%, *n* = 7; junior/3rd year = 25.9%, *n* = 7; senior/4th year = 14.8%, *n* = 4), and living in on-campus housing (96.3%, *n* = 26). Student-athletes engaged in three different sports: soccer (33.3%, *n* = 9), volleyball (44.4%, *n* = 12), and basketball (22.2%, *n* = 6) and were actively participating in practices and games at least 3 days per week.

### 2.2. Instruments

#### 2.2.1. Basic Demographic Information

Basic personal and demographic information including age, sex, ethnicity (i.e., White, Black/African American, Hispanic, Asian, Multi-ethnic), academic status (i.e., freshman/1st year, sophomore/2nd year, junior/3rd year, senior/4th year, etc.), sport, self-reported height, and weight (i.e., current, lowest, highest, and ideal) were collected. A specific health history questionnaire was utilized to screen for exclusion factors.

#### 2.2.2. Anthropometric Measures

Basic anthropometric measures, including height, weight, and body composition, were conducted in accordance with the American College of Sports Medicine (ACSM) testing procedures. Height was measured using a stadiometer (Shorr Productions, Olney, MD, USA) to the nearest 0.1 cm. Weight was measured wearing minimal clothes to the nearest 0.01 kg with a scale (Tanita, 331S, Tokyo, Japan). Body composition was assessed using a Tanita scale (Tanita SC-331S Body Composition Scale, Tanita Co., Tokyo, Japan) and Dual-Energy X-Ray Absorptiometry (DXA; Lunar Prodigy, GE Healthcare, Madison, WI, USA; Horizon A, Hologic Inc., Marlborough, MA, USA).

#### 2.2.3. Resting Metabolic Rate

The MedGem (Microlife Home Solutions Inc., Golden, CO, USA) was used to calculate resting metabolic rate (RMR) via indirect calorimetry. The MedGem is a clinically validated handheld and portable measurement device that measures oxygen consumption (VO_2_) to determine RMR with an interclass reliability ranging between 0.91 and 0.97 [44]. RMR ratio is the ratio of an individual’s measured RMR to their predicted RMR. We used the Harris–Benedict equation to predict RMR [45]. Using the Harris–Benedict equation, previous literatures have noted a RMR ratio ≤ 0.90 as a proxy for categorizing exercising females as energy deficient [46,47,48,49,50].

#### 2.2.4. Energy Availability

Energy availability is defined as the dietary energy remaining after exercise and was determined by subtracting the energy expended by metabolic demand of exercise, also known as EEE, from EI. Energy availability is expressed as EI—EEE in kilocalories per kilogram of FFM). An EA of ≤30 kcal/kg FFM was defined as LEA [15].

#### 2.2.5. Energy Intake

Participants utilized the online Food Prodigy program (ESHA food processor 8.0, Salem, OR) during the 7-day data collection period to self-report EI. Participants were briefed on portion size and given examples for their food log descriptions. Dietary logs were examined for total caloric intake (kilocalories) and macronutrient intake (proteins, carbohydrates, and fats). Macronutrient requirements were assessed using the daily needs for fuel and recovery guidelines by the ACSM and the Academy of Nutrition and Dietetics, Dietitians of Canada (carbohydrates 6–10 g/kg/day, protein 1.2–2 g/kg/day, fats 20–35% of total caloric intake, and saturated fat no more than 10% of total caloric intake) [51]. Participants were considered highly active if they engaged in 1–3 h/day of moderate to high-intensity exercise.

#### 2.2.6. Total Daily Energy Expenditure (TDEE)

Total Daily Energy Expenditure is the total amount of calories expended by one person in a 24 h period and is composed of 3 major components: (1) RMR, (2) thermic effect of feeding, and (3) thermic effect of activity. Polar Ignite heart rate (HR) monitors (Polar Electro Inc., Bethpage, NY, USA) were used to estimate TDEE across 7 days. These devices are a non-invasive method of continuously monitoring the individual wearing the device for TDEE and EEE. Wrist-worn devices including those by Polar Electro Inc, have been determined to accurately assess HR at rest and during moderate activity as compared to the gold standard, electrocardiogram (r ≥ 0.90, standard error of estimate ≤5 bpm) [52].

#### 2.2.7. Exercise Energy Expenditure

Exercise energy expenditure was calculated using the Polar Ignite or Polar H10 heart rate monitor (Polar Electro Inc., Bethpage, NY, USA). The watch calculates metabolic equivalents (METs) during exercise, derived from the participant’s HR and the corresponding percent of maximal oxygen consumption ($\overset{\cdot }{V}$O_2max_). Participants were instructed to wear the watch as much as possible and to use the watch’s exercise function specifically to record planned and intentional exercise. During activities where the wrist-worn devices were not appropriate, such as games, the participants used the Polar H10 chest strap to measure HR and estimate EEE.

#### 2.2.8. Eating Disorder Inventory 3

The Eating Disorder Inventory 3 (EDI-3) was used to assess ED risk. The EDI-3 is a validated tool with high test–retest reliability (0.98) for ED risk and a general psychological maladjustment coefficient of 0.97. The EDI-3 is made up of 91 items across 3 ED-specific scales: (1) drive for thinness, (2) bulimia, and (3) body dissatisfaction, and 9 general psychological scales: (1) low self-esteem, (2) personal alienation, (3) interpersonal insecurity, (4) interpersonal alienation, (5) interceptive deficits, (6) emotional dysregulation, (7) perfectionism, (8) asceticism, and (9) maturity fear [53]. Participants responded to all questions on a 6-point Likert scale (always, usually, often, sometimes, rarely, and never) to determine ED behaviors and associated psychological behaviors. The 12 scales yield 6 composite scores: 1 ED specific (ED risk composite) and 5 general integrative psychological constructs (ineffectiveness, interpersonal problems, affective problems, overcontrol, and the overall psychological maladjustment composite).

Permission to use the EDI-3 is granted with the purchase and a computer-based scoring program was used to assess the participants’ outcomes. Individualized score reports with raw scores, T scores, percentiles, and qualitative classifications (low clinical, typical clinical, elevated clinical) for all EDI-3 scales were generated by the software. Clinical ranges are based on percentile ranges for the U.S. Adult Combined Clinical sample (i.e., low clinical = 1st to 24th percentile, typical clinical = 25th to 66th percentile, and elevated clinical = 37th to 99th percentile). Participants with scores of typical clinical or elevated clinical on two or more composites are considered “at risk” for an ED.

#### 2.2.9. Eating Disorder Inventory-3 Symptoms Checklist

The EDI-3 Symptoms Checklist (EDI-SC) was used in conjunction with the EDI-3 to identify participants at risk for EDs based on pathogenic behaviors to control weight (i.e., binge eating, self-induced vomiting, exercise patterns, and the use of laxatives, diet pills, and diuretics). The number of items in the inventory ranges based on reporting yes or no to behaviors. For each question answered as “yes,” the participant had follow-up questions regarding the frequency of behaviors. Participants who met the criteria for at least one pathogenic behavior were considered “at-risk” for EDs using the EDI-SC.

### 2.3. Procedures

#### 2.3.1. Recruitment and Screening

Participants were recruited from 3 HBCUs in the Southeast U.S. via their athletic trainers. The primary investigator hosted a formal recruiting meeting with the teams to provide an overview of the research study. Contact information was collected during these meetings, and further information detailing the research study’s purpose, protocol, risks, and benefits was emailed to interested participants. Participants received a copy of the IRB-approved consent form to review and a brief survey to screen for any exclusion criteria. Participants who met the inclusion criteria were directed to the second portion of the web-based survey.

#### 2.3.2. Data Collection

Participants completed anthropometric measurements, RMR assessments, and the EDI-3 and EDI-SC. Participants were briefed on how to properly utilize the Polar Ignite HR monitor and how to log their exercise and dietary intake in Food Prodigy (Version 2.2.0, ESHA software, Salem, OR, USA). Data were collected for a total of 7 consecutive days per participant. The Polar Ignite monitor was worn continuously, and the physical activity tracker was turned on during all planned and intentional hours. The Polar H10 chest HR monitors were set up and started by a research team member prior to games or practices where the wrist-worn monitor was not allowed. In-person reminders were given or sent via text messages to the participants to complete all exercise and food logs each day. Following the completion of the 7 consecutive days, participants returned all research materials to the primary investigator.

### 2.4. Data Analysis

Data were analyzed using SPSS statistical software (Version 29; SPSS Inc., Armonk, NY, USA) with a significance level set at *p* < 0.05. Basic descriptive statistics (mean and standard deviation) were used for all demographic information, RMR, TDEE, EEE, and EA. Frequencies and percentages were calculated to determine the prevalence of LEA, ED risk, and pathogenic behaviors. Cross-tabulations were used to determine the difference between participants with LEA with or without ED risk.

## 3. Results

### 3.1. Demographics and Anthropometric Measures

Self-reported demographics and anthropometric measures are presented in Table 1.

### 3.2. Energy Needs

Across all participants, the average EI was 1461.0 ± 451.2 kcals and average TDEE was 2712.5 ± 545.6 kcals, resulting in a mean negative energy balance of −1251.5 ± 674.7 kcals. Ten participants (37.0%) did not meet the intake for their RMR. Furthermore, the average EEE was 718.97 ± 194.5 kcals and EA was 15.9 ± 10.1 kcals/kg/FFM/day, yielding a total of 92.6% (*n* = 25) of participants below the 30 kcal/kg/FFM/day threshold for LEA, and 7.4% (*n* = 2) had sub-optimal EA between 30 and 45 kcal/kg/FFM/day. Variables related to energy needs are depicted in Table 2.

The recommended carbohydrate intake (6–10 g/kg/day) was not met by any of the participants, and only 14.8% (*n* = 4) were within the recommended protein intake values (1.2–2 g/kg/day). All participants consumed the minimally recommended fat intake (20–35% kcals), with 44.4% (*n* = 12) exceeding the 35% threshold. When examining saturated fats, 63% (*n* = 17) of the participants consumed above the recommended 10% of all caloric intake.

### 3.3. Eating Disorder Risk

Overall, ED risk using the EDI-3 and/or the EDI-SC was found in 59.3% (*n* = 16) of the female student-athletes. Table 3 characterizes the EDI-3 primary scales and composite scores. The typical clinical category reflects scores and characteristics observed in patients diagnosed with an ED. Female student-athletes primarily endorsed low clinical and elevated clinical scores in the ED risk scales. A larger percentage of typical clinical and elevated clinical scores were present across the interpersonal insecurity (74.1%, *n* = 20) and interpersonal alienation (70.4%, *n* = 19) scales, which make up the interpersonal problems composite (70.4%, *n* = 19), emotional dysregulation scale (55.6%, *n* = 15), perfectionism scale (92.6%, *n* = 25), and maturity fears scale (81.5%, *n* = 22). Furthermore, female student-athletes in this sample thought they would weigh 3.2 ± 4.4 kg more if they did not consciously/mentally try to control their weight, and their ideal weight was 2.1 ± 4.9 kg less than their current weight.

Bivariate correlations are presented in Table 4. Personal alienation had a significant positive relationship with low self-esteem (*r* = 0.863, *p* < 0.001). Interpersonal insecurity had significant positive relationship with low self-esteem (*r* = 0.531, *p* = 0.004) and personal alienation (*r* = 0.623, *p* < 0.001). Interpersonal alienation had a significant relationship with low self-esteem (*r* = *0*.562, *p* = 0.002), personal alienation (*r* = 0.707, *p* < 0.001), and interpersonal insecurity (*r* = 0.623, *p* < 0.001). Interoceptive deficits had a significant positive relationship with low self-esteem (*r* = 0.543, *p* = 0.003), personal alienation (*r* = 0.681, *p* < 0.001), interpersonal insecurity (*r* = 0.563, *p* = 0.002), and interpersonal alienation (*r* = 0.766, *p* < 0.001). Emotional dysregulation had a significant positive relationship with low self-esteem (*r* = 0.546, *p* = 0.003), personal alienation (*r* = 0.699, *p* < 0.001), interpersonal insecurity (*r* = 0.464, *p* = 0.015), interpersonal alienation (*r* = 0.641, *p* < 0.001), and interoceptive deficits (*r* = 0.697, *p* < 0.001). Asceticism had a significant positive relationship with low self-esteem (*r* = 0.389, *p* = 0.045), interoceptive deficits (*r* = 0.437, *p* = 0.023), and emotional dysregulation (*r* = 0.628, *p* < 0.001). Maturity fears had a significant positive relationship with low self-esteem (*r* = 0.475, *p* = 0.012) and personal alienation (*r* = 0.447, *p* = 0.019).

The EDI-SC was used to examine the risk for pathogenic behaviors (i.e., dieting, excessive exercise, binge eating, purging, use of diet pills/diuretics/laxatives, etc.); findings are presented in Table 5. A total of 14 participants (51.9%) engaged in pathogenic behaviors to control their weight. Further assessment of the EDI-SC demonstrated that one participant (3.7%) engaged in three pathogenic behaviors, 18.5% (*n* = 5) engaged in two pathogenic behaviors, and 29.6% (*n* = 8) engaged in one pathogenic behavior. Participants did not report using laxatives, diet pills, or diuretics to control their weight.

### 3.4. Low Energy Availability with or Without Eating Disorder Risk

Of the 25 participants identified with LEA, 60% (*n* = 15) had LEA with ED risk, while 37.0% had LEA without ED risk. Interestingly, of the two participants without LEA, 3.7% (*n* = 1) was at risk for an ED using both the EDI-3 and the EDI-SC.

## 4. Discussion

Low EA has been examined widely across female student-athletes, with varying findings across sports [17,18,19,21,22,23,37,38,39,40,42,54,55]. However, demographic information as it pertains to race and ethnicity is often not disclosed or explored in many studies. There is limited research among athletes of color, making it challenging for results to apply across all populations, specifically communities that have been underrepresented and marginalized, such as student-athletes at HBCUs. To the best of our knowledge, this is the first study to specifically examine energy needs and risk of LEA with or without ED risk in female student-athletes attending HBCUs. As hypothesized, our findings suggest female student-athletes do not meet the recommended dietary intake to meet the demands of their activity, resulting in an increased prevalence of LEA. Additionally, female student-athletes in this group display a high risk for EDs at concerning rates.

### 4.1. Energy Expenditure and Energy Intake

Exercise energy expenditure and EI were objectively measured in 27 female student-athletes over seven consecutive days during in-season play. Overall, mean EEE was comparable to previous studies across NCAA Division I and II female collegiate student-athletes, where EEE ranges are reported between 600 to over 1000 kcals/day [22,23,41,42,56]. While comparisons across sports were not made, the soccer student-athletes in the present study had lower EEE than those in volleyball and basketball. This may be due to multiple competitions during the seven consecutive days of data collection for the latter teams.

On average, participants self-reported consuming 1461.0 ± 451.2 kcals/day, which is below that previously reported in females in team sports [41,42,54,55,57]. Reed et al. [39] noticed female soccer players reported lower EI during meals where they were responsible for preparing or purchasing, which most often occurs during mid-season. Their findings could potentially be a factor for the student-athletes in this study, as they were also mid-season during data collection and primarily responsible for attaining their own meals while also being limited by the options provided on campus. We should also note most of the participants lived in on-campus dormitories, potentially limiting their meals to items purchased or attained in the on-campus dining facilities, which are for all students at their institutions and have limited hours.

To examine the relative macronutrient intake by participants, we utilized the ACSM and Academy of Nutrition and Dietetics, Dietitians of Canada guidelines for appropriate types and amounts of dietary intake [51]. These were developed to promote optimal health and performance across training and competitive sports and make the recommendation that athletes who engage in 1–3 h a day of moderate to high-intensity exercise should consume 6–10 g/kg/day of carbohydrates, 1.2–2 g/kg/day of protein, and 20–35% of fats with no more than 10% of total caloric intake coming from saturated fats [51]. Through the years, experts have debated the importance, role, and contribution of each macronutrient in athletes’ nutrition, with one factor remaining consistent. Carbohydrates are an indispensable need in an athlete’s diet and are the macronutrient most efficiently metabolized by the body to provide energy during high-intensity training [58]. Unfortunately, throughout nutrition research, carbohydrate intake continues to be deficient relative to weight and energy expenditure in athletes. Reed et al. [56] and Beermann et al. [20] reported, respectively, that 57.9% and 85% of their female participants did not meet carbohydrate recommendations during their competitive seasons. These studies along with our findings (100%, *n* = 27) demonstrate that this continues to be a problem and can be due to the restriction of total EI. Exploration of relative macronutrient intake across female sports has shown slightly higher carbohydrate averages than those in the present study (cross-country = 4.7 ± 1.9 g/kg/d [20], basketball = 3.7 ± 0.7 g/kg/d [42], soccer = 3.7 ± 1.0 g/kg/d [54], lacrosse = 3.6 ± 1.1 g/kg/d [41]); however, female and male collegiate swimmers with LEA have similar findings with a relative carbohydrate intake of 2.8 ± 0.5 g/kg/d [38].

Protein is considered the primary macronutrient to support muscle repair and rebuilding, and there appears to be a greater compliance for the recommended intake. On average, previous studies show relative protein intake ranging from 1.1 to 2.0 g/kg/d [21,38,41,42,54], which aligns closely with the recommended dietary intake guidelines [51]. In comparison, our findings indicated that 85.2% (*n* = 23) of female athletes were under the recommendations for protein intake, with an average relative intake of 0.89 ± 0.35 g/kg/day. Given the limited consumption of carbohydrates in these female student-athletes, protein intake becomes especially important to maintain energy levels, preserve muscle mass, and perform essential functions of the body.

In comparison to protein and carbohydrate dietary intake, Thomas and colleagues [51] recommended fat intake meets the public health guidelines, which are based on percentages of total dietary intake and not g/kg of body weight. Consistent with previous studies [20,38,56], our findings indicate an elevated proportion of dietary intake (% of total kcal = 35.2 ± 4.7) is derived from fats [20,38,56]. We noted that 44.4% of participants surpassed the 35% threshold for fats and 63% above the recommended 10% of kcal from saturated fats. These findings are not uncommon among athletes, where 49% of cross-country runners reported consuming more than 35% of kcal from fats and over 50% consumed more than 10% of kcal from saturated fats [20].

### 4.2. Eating Disorder Risk

We examined ED risk using a multidimensional approach. The combination of the EDI-3 and the EDI-SC resulted in an overall risk of 59.3%. Recently, Torres-McGehee et al. [33], documented ED risk among female student-athletes participating in ball or team sports (i.e., basketball, soccer, softball, volleyball, and beach volleyball) at 21.9% (*n* = 87/397) using the Eating Attitudes Test 26 (EAT-26) questionnaire. While using a different assessment tool and a smaller sample size, our findings nearly triple the prevalence of ED risk previously found in female ball or team student-athletes, which may be due to the assessment of psychological factors associated with ED in the EDI-3 as compared to the EAT-26, which solely focuses on eating attitudes and behaviors. Furthermore, the risk for EDs among female athletes across all sports ranged between 9.5 and 47.3% through the years, comparatively less than our sample [33,59,60,61,62]. While no female athlete scored in the typical clinical range for the ED risk composite, we found a small number of female student-athletes in the typical clinical scales for drive for thinness (14.8%) bulimia (11.1%) and body dissatisfaction (7.4%), with three student-athletes scoring in that category in two of the three scales. Moreover, our findings are higher than previous research focused on ED risk in racial and ethnically diverse students, where 21 to 41.9% of women of color (Black = 22%, Hispanic = 31.4%) were at risk using the five-item SCOFF screening tool [63]. The SCOFF questionnaire primarily focuses on the core features of anorexia nervosa and bulimia nervosa. In comparison, the EDI-3 utilized nine psychological scales associated with EDs, which could be the reason for the prevalence in our sample is much higher.

#### 4.2.1. Psychological Traits

Psychological components and personality traits make up some of the risk factors and/or comorbidities of DE and clinical EDs. Evidence suggests there is an interaction between personal, environmental/cultural, and genetic factors to make EDs a multidimensional mental disorder [64]. When examining ED risk using the comorbid psychological composites, we found an increased risk in the interpersonal problems, affective problems, interpersonal problems, affective problems, and over control composites with 70.4%, 37%, and 40.7% of participants scoring in the typical clinical and elevated clinical ranges, respectively. Most female student-athletes presented with high risk in the maturity fears scale, included in the general psychological maladjustment composite, which considers all nine psychological scales.

The interpersonal problems composite is made up of two scales: interpersonal insecurity and interpersonal alienation. Most of the female student-athletes in this sample scored within the typical clinical and elevated clinical risk in this composite. In comparison, previous studies across female student-athletes have a prevalence of typical clinical and elevated clinical risk ranging between 36.4 and 57.9% and 21.1 and 35.5% for interpersonal insecurity and interpersonal alienation, respectively [33,40]. As a composite, Garner et al. [53] states that higher scores indicate respondents may experience significant to extreme distress and social relationships may be tense, insecure, disappointing, unrewarding, and of poor quality. Furthermore, interpersonal problems play a significant role in the development and maintenance of EDs. Previous studies demonstrate an association between interpersonal difficulties (i.e., being too friendly, non-assertive, and too cold) and treatment outcomes, as patients may not engage with therapists at a meaningful or functional level [2,65].

The affective problems composite examines one’s ability to identify, understand, and respond to emotional states correctly through the interoceptive deficits and emotional dysregulation scales [53]. The latter explores tendencies towards mood instability and self-destructive behavior, such as substance abuse. More than half of our participants displayed typical clinical or elevated clinical risk for the emotional dysregulation scale, significantly higher than the risk seen across female student-athletes from predominately white institutions [33,40]. As college students, student-athletes transition to a new environment from adolescence to independence and adulthood; this transition comes with changes to perception, social support systems, and academic and athletic stressors that may create a vulnerable period for substance use and abuse as coping mechanisms.

Fairburn’s [66] transdiagnostic theory of EDs established high perfectionism as a core trait of the condition. Perfectionism is explored in the EDI-3 as part of the over control composite and looks at an individual’s concern with striving for flawlessness accompanied by the drive to meet high standards and the expectations set forth by themselves, others, or both [53]. For student-athletes specifically, this can present as a relentless pursuit to meet the expectations of coaches, teammates, and family. Many times, athletes may see perfectionism as a strength because it demonstrates their dedication to the sport; however, perfectionism can be a debilitating factor to their mental health [67]. We found that over 90% of female student-athletes are at typical clinical and elevated clinical risk of perfectionism. While types of perfectionism were not explored, previous studies have found self-critical perfectionism and socially prescribed perfectionism as predictive variables of ED risk [68,69]. Furthermore, for women of color, perfectionism has been positively correlated with DE cognitions [70]. The desire to be perfect among student-athletes and women of color may cause increased feelings of negative self-worth, negative emotions, stress, and anxiety, all of which can contribute further to ED risk.

The maturity fear scale had the second highest prevalence of typical clinical and elevated clinical risk at 81.5%. This scale is described as wanting to return to the safety of childhood and avoiding the developmental demands and increased weight of adulthood [53]. Across the literature maturity fears among college student-athletes and artists continue to be elevated, ranging from 59.5 to 77.5% for combined typical clinical and elevated clinical risk [23,40,71]. Reluctancy toward maturity and fear toward the typical aging process seem to be emerging across young adults, specifically undergraduate students. Beginning in the early 2000s, Smith et al. [72] explored various scales of the EDI-3 in undergraduate women and found mean scores for maturity fears gradually increased through the years. The authors stated a concern with maturity fear is how difficult it can make aging for young adults and the impact it may have to their psychological well-being [72]. Similarly, aging may be a factor for patients with ED pathologies. They may also place additional focus on the physical changes their bodies are going through and face concerns of body dissatisfaction or body dysmorphia. Findings vary with regard to body satisfaction among Black women. While early investigations demonstrated a positive body image [73], more recent studies observed body dissatisfaction levels similar to those of White women, specifically in early adulthood, which corresponds to the transition into college years [74].

#### 4.2.2. Pathogenic Behaviors

While racial and ethnically diverse women may not have lifetime prevalence rates as high as White/Caucasian women, they engage in multiple pathogenic behaviors to control weight. Perez et al. [75] used the Eating Disorder Examination Questionnaire to explore ED symptomology among racial and ethnically diverse women, with subclinical level symptoms to determine the central ED symptoms in this population. Black, Hispanic, and Asian American women engaged in various pathogenic behaviors at alarming rates (binge eating = 59.4%, vomiting = 74%, laxative misuse = 76.9%, compulsive exercise = 76.9%, limiting food = 68.6%, fasting = 51.1%, and rules of eating = 61.7%). The central symptoms that emerged from their study were weight concerns (i.e., strong desire to lose weight and fear of weight gain), which were consistent with the transdiagnostic theory of EDs, where weight remains one of the core psychopathologies of EDs [66]. Similarly, when examining mental and ideal weight across our sample, we noted participants perceived themselves larger than they are and ideally wished to be smaller. It should be noted that 66.7% of female student-athletes reported engaging in excessive exercise to control weight, which was the behavior most commonly noted by Perez et al. [75].

### 4.3. Low Energy Availability with or Without Eating Disorder Risk

We demonstrate a high prevalence of LEA with and without ED risk in female student-athletes of color from HBCU institutions. Low dietary intake can be regarded as the influential factor to LEA; however, females in this sample actively engage in pathogenic behaviors to control their weight and have an increased risk for psychological factors highly associated with ED pathologies. We noted that of the participants with LEA, 60% presented with ED risk, aligning with findings from previous studies where the prevalence ranged from 52.6% to 76% [23,40]. To date, LEA prevalence has been explored across sports and the performing arts, using objective measures and predictive questionnaires. Across outdoor field sports (soccer and lacrosse), LEA prevalence has ranged from 16% (specifically in the soccer post-season) to as high as 66.7% [23,41,54,55]. Interestingly, those rates are higher than those observed across female athletes from individually-focused sports such as cross-country (41%) [20], swimming (40%) [38], and track and field (52%) [22]. However, both outdoor field and individual sport groups have a prevalence of LEA less than observed in the current sample. We should also note that of the above-mentioned studies, only two reported racial or ethnic demographics of participants, and no information is provided beyond the distribution [20,22].

To the best of our knowledge, limited studies exist depicting LEA prevalence across student-athletes from diverse racial and ethnic backgrounds. Ackerman et al. [37] utilized ED and DE questionnaires as a surrogate for LEA. While the sample was primarily Caucasian female athletes, findings of LEA were reported across race/ethnicity (Black = 4.2%, Hispanic = 5.7%, Asian = 5.5%, Pacific Islander = 0.2%, and Other = 3.2%). A second study by Marzuki et al. [76] used an online questionnaire and reported that 67.2% of athletes in Malaysia had medium to high REDs risk; females specifically accounted for 41.2%. Our study is unique in comparison, as we used objective measures to assess EA and determined an elevated risk of LEA in a diverse population.

### 4.4. Limitations and Future Research

While this is a novel study, given the focus on an understudied diverse population, we must acknowledge the limitations. This was a small convenient sample where participants were only recruited from the Southeast U.S. A larger sample across different geographic areas would provide further details on LEA prevalence with or without ED risk. We assume participants completed all questionnaires accurately and honestly. We utilized the EDI-3 and EDI-SC as a multidimensional approach to ED risk; however, we should note that factor analysis on the EDI-3 has a mediocre fit in the African American samples [77]. To date, the literature indicated that ED screening and diagnostic tools are increasingly culture-bound and biased, often overlooking marginalized racial and ethnic groups. Estimates of EA were derived from commercial HR monitors and self-reported dietary logs which are subject to bias due to under-reporting or purposeful alteration to fit the norm. However, to mitigate inaccuracy we used 7-day dietary plan to minimize daily bias [78], we also advised and provided physical copies of portion examples, and we were available during the entirety of the study to help input data and answer questions about their dietary logs. Lastly, no statistically significant conclusions can be drawn between participants with LEA with or without ED risk as the cell count assumption for the chi-square analysis was violated in the statistical analysis.

Both the Triad Coalition and IOC acknowledge the need for studies with racial and ethnically diverse samples [79]. While we provide some insight into this population, more studies are needed to determine how LEA impacts athletes of color and to increase awareness among healthcare providers about the associated psychological traits in racial and ethnic women. Future research should focus on the examination of EA with or without ED risk across HBCU male student-athletes, as well as examining energy needs and EA across different time points such as pre-, mid-, and post-season across both female and male student-athletes at HBCUs. Moreover, additional physiological markers such as blood biomarkers, immunological factors, and bone mineral density should be explored as they may provide insight into the different components of the female Triad and REDs.

## 5. Conclusions and Practical Applications

In summary, our results indicated that HBCU female student-athletes displayed LEA and increased ED risk with the majority showing signs of both. These findings highlight the need for targeted interventions to improve EA and support mental health within this underserved population. Additionally, we note low EI, specifically low carbohydrate intake, as a primary factor influencing LEA. To improve the safety and reduce injury and illness risk among these student-athletes, clinicians working with this community should prioritize education on optimal fueling and balanced diet, emphasizing the importance of optimal EA, balanced diets and the roles of carbohydrates and protein in fueling performance and recovery. In addition, the availability of sports dietitians is encouraged to provide student-athletes with individualized nutrition plans considering their specific needs, training schedules, and dietary preferences to ensure optimal fueling.

Moreover, the use of pathogenic behaviors and increased psychological risk factors were primarily identified across female student-athletes at risk for EDs. While EDs remain prevalent among White females, they also manifest across ethnically diverse populations, and psychological signs and symptoms may present first. Early identification of ED behaviors and particularly associated psychological signs is imperative to mitigate long-term health, student-athletes should also be supported with mental health resources, such as counseling services and support groups, tailored to their specific needs, and emphasizing the connection between nutrition, mental health, and athletic performance.

Currently, limited research on LEA and ED risk exists representing female student-athletes of color; therefore, although this observational study involved a small sample of HBCU female student-athletes (*n* = 27), it is the first to explore these factors in the United States. Student-athletes come from diverse racial and ethnic backgrounds; hence, it is imperative that research includes a representative sample to ensure the findings are inclusive and applicable to all student-athletes.

## Figures and Tables

**Table 1:** Self-reported and physical measurements for female student-athletes (*n* = 27). Values are presented in mean ± standard deviation.

Demographic		Mean ± SD
Age (years)		19 ± 1
Height (cm)		166.9 ± 7.6
Weight (kg)		68.8 ± 11.3
	Self-Reported Highest	71.2 ± 11.8
	Self-Reported Lowest	63.6 ± 9.5
	Self-Reported Mental ^a^	71.3 ± 9.4
	Self-Reported Ideal	66.1 ± 7.6
	Current—Self-Reported Mental	−3.2 ± 4.4
	Current—Self-Reported Ideal	2.1 ± 4.9
Body Mass Index (kg/m^2^)		24.6 ± 3.1
Free Fat Mass (kg)		49.3 ± 5.8
Body Fat (%)		27.9 ± 4.9

^a^ Mental weight: Individual’s perceived weight if one does not consciously try to control their weight.

**Table 2:** Energy needs assessment presented as mean ± standard deviation or percent (frequency).

			Mean ± SD
**Energy Needs**			
	Resting Metabolic Rate (RMR; kcals)		1311.1 ± 228.5
	Harris–Benedict Equation RMR (kcals)		1531.6 ± 116.0
	RMR Ratio		0.85 ± 0.12
	Energy Intake (kcals)		1461.0 ± 451.2
	Exercise Energy Expenditure (kcals)		718.97 ± 194.5
	Energy Availability (kcals/kg FFM)		15.9 ± 10.1
	Total Daily Energy Expenditure (kcals)		2712.5 ± 545.6
	Energy Balance (kcals)		−1251.5 ± 674.7
**Macronutrients**			
	Proteins (g/day)		58.8 ± 18.2
	Relative Protein (g/kg/day)		0.89 ± 0.35
	Within Recommendations		14.8% (4)
	Under Recommendations		85.2% (23)
	Carbohydrates		180.9 ± 71.8
**Relative Daily Macronutrient Intake**			
	Relative Carbohydrates (g/kg/day)		2.67 ± 1.0
		Under Recommendations	100% (27)
	Fats (g/day)		509.7 ± 147.9
	Relative Fats (% of total kcal/day)		35.2 ± 4.7
		Above Recommendation	44.4% (12)
		Within Recommendations	55.6% (15)
	Saturated Fat		157.9 ± 56.2
	Relative Saturated Fats (% of total kcal/day)		10.8 ± 2.2
		Above Recommendation	63.0% (17)
		Below Recommendation	37.0% (10)

Abbreviations: FFM, fat free mass.

**Table 3:** Eating Disorder Characteristics presented as mean and standard deviation; percent and frequency.

		Raw Score	Low Clinical	Typical Clinical	Elevated Clinical
		Mean ± SD	% (*n*)	% (*n*)	% (*n*)
**Eating Disorders Risk Scales**					
	Drive for Thinness	6.3 ± 6.9	85.2 (23)	14.8 (4)	0 (0)
	Bulimia	1.7 ± 1.9	88.9 (24)	11.1 (3)	0 (0)
	Body Dissatisfaction	10.6 ± 7.2	95.6 (25)	7.4 (2)	0 (0)
**Psychological Scales**					
	Low Self-Esteem	3.4 ± 4.5	85.2 (23)	14.8 (4)	0 (0)
	Personal Alienation	6.4 ± 5.5	77.8 (21)	11.1 (3)	11.1 (3)
	Interpersonal Insecurity	11.4 ± 5.5	25.9 (7)	48.1 (13)	25.9 (7)
	Interpersonal Alienation	7.9 ± 3.8	29.6 (8)	59.3 (16)	11.1 (3)
	Interoceptive Deficits	8.7 ± 7.5	70.4 (19)	22.2 (6)	7.4 (2)
	Emotional Dysregulation	5.0 ± 4.6	44.4 (12)	40.7 (11)	14.8 (4)
	Perfectionism	14.4 ± 4.5	7.4 (2)	66.7 (18)	25.9 (7)
	Asceticism	4.8 ± 3.8	88.9 (24)	11.1 (3)	0 (0)
	Maturity Fears	11.0 ± 4.8	18.5 (5)	48.1 (13)	33.3 (9)
**Composite**					
	Eating Disorder Risk Composite	98.8 ± 17.1	100 (27)	0 (0)	0 (0)
	Ineffectiveness Composite	70.6 ± 19.8	85.2 (23)	11.1 (3)	3.7 (1)
	Interpersonal Problems Composite	94.0 ± 15.9	29.6 (8)	55.6 (15)	14.8 (4)
	Affective Problems Composite	84.2 ± 15.9	63.0 (17)	29.6 (8)	7.4 (2)
	Over control Composite	87.0 ± 11.0	59.3 (16)	37.0 (10)	3.7 (1)
	General Psychological Maladjustment	389.2 ± 49.8	66.7 (18)	25.9 (7)	7.4 (2)

For all scales and composites, high scores reflect greater distress.

**Table 4:** Zero-order Correlations.

Variable	1.	2.	3.	4.	5.	6.	7.	8.	9.
1. Low Self-Esteem	**-**								
2. Personal Alienation	**0.863**	**-**							
3. Interpersonal Insecurity	**0.531**	**0.623**	**-**						
4. Interpersonal Alienation	**0.562**	**0.707**	**0.623**	**-**					
5. Interoceptive Deficits	**0.543**	**0.681**	**0.563**	**0.766**	**-**				
6. Emotional Dysregulation	**0.546**	**0.699**	**0.464**	**0.641**	**0.697**	**-**			
7. Perfectionism	0.292	0.337	0.075	0.374	0.332	0.304	**-**		
8. Asceticism	**0.389**	0.270	0.053	0.228	**0.437**	**0.628**	0.185	**-**	
9. Maturity Fears	**0.475**	**0.447**	0.161	0.113	0.142	0.180	0.020	0.245	**-**

Bold text indicates *p* < 0.05.

**Table 5:** Distribution of LEA, ED, and LEA with ED risk.

		% (*n*)
**LEA Risk**		92.6 (25)
**ED Risk**		59.3 (16)
	EDI-3	7.4 (2)
	EDI-SC	11.1 (3)
	Both EDI-3 and EDI-SC	40.7 (11)
**LEA with ED Risk**		60.0 (15/25)
**Pathogenic Behaviors**		
	Dieting	40.7 (11)
	Binging	18.5 (5)
	Purging	11.1 (3)
	Laxatives	0 (0)
	Diet Pills	0 (0)
	Diuretics	0 (0)
**Exercise to Control Weight**		
	0% of time	33.3 (9)
	<25% of time	33.3 (9)
	25–50% of time	25.9 (7)
	More than 50% of time	7.4 (2)
	100% of time	0 (0)

Abbreviations: EA, energy availability; ED, eating disorder; EDI, Eating Disorder Inventory; LEA, low energy availability; SC, Symptom Checklist.

## Data Availability

The raw data supporting the conclusions of this article will be made available by the authors, without undue reservation.
